# Acoustic signalling reflects personality in a social mammal

**DOI:** 10.1098/rsos.160178

**Published:** 2016-06-29

**Authors:** Mary Friel, Hansjoerg P. Kunc, Kym Griffin, Lucy Asher, Lisa M. Collins

**Affiliations:** 1 School of Biological Sciences, Queen's University Belfast, Medical Biology Centre, Belfast BT9 7BL, UK; 2 School of Animal, Rural and Environmental Sciences, Nottingham Trent University, Burton Street, Nottingham NG1 4BU, UK; 3 Centre for Behaviour and Evolution, Institute of Neuroscience, Newcastle University, Newcastle upon Tyne NE1 7RU, UK; 4 School of Life Sciences, University of Lincoln, Brayford Pool, Lincoln LN6 7TS, UK

**Keywords:** acoustic signalling, animal personality, pig, environmental enrichment

## Abstract

Social interactions among individuals are often mediated through acoustic signals. If acoustic signals are consistent and related to an individual's personality, these consistent individual differences in signalling may be an important driver in social interactions. However, few studies in non-human mammals have investigated the relationship between acoustic signalling and personality. Here we show that acoustic signalling rate is repeatable and strongly related to personality in a highly social mammal, the domestic pig (*Sus scrofa domestica*). Furthermore, acoustic signalling varied between environments of differing quality, with males from a poor-quality environment having a reduced vocalization rate compared with females and males from an enriched environment. Such differences may be mediated by personality with pigs from a poor-quality environment having more reactive and more extreme personality scores compared with pigs from an enriched environment. Our results add to the evidence that acoustic signalling reflects personality in a non-human mammal. Signals reflecting personalities may have far reaching consequences in shaping the evolution of social behaviours as acoustic communication forms an integral part of animal societies.

## 1. Introduction

Acoustic communication plays a central role in various aspects of animals' life history from mate attraction and territory defence to parental care and anti-predator behaviour in many species [1]. As such, acoustic signals convey a wide range of information about the signaller, including their emotional, motivational and physiological state [2–4]. There is increasing interest in discovering how the social environment affects the evolution and maintenance of consistent behavioural variation between individuals of the same species [5]. As acoustic signalling plays an integral role in interactions in animal societies, investigating vocalizations is particularly relevant for answering questions about the effect of interactions on individual behavioural variation.

Animal personalities, also known as ‘behavioural syndromes’ [6] or ‘coping styles’ [7] are defined as consistent individual differences in behaviour across situations and time [6] and they may have consequences for fitness [8,9]. The way in which individuals react to environmental stressors has been studied from the perspective of coping styles and these are characterized by consistent individual behavioural and physiological traits termed ‘reactive’ and ‘proactive’ coping styles’ [7]. Acoustic signals form part of many species' response to stressors, for example, alarm calling when a threat is perceived or using contact calls when an individual is separated from their social group [10,11]. The repeatability of acoustic signalling rate and acoustic parameters in mammals has received some attention in the literature [11–13], with many finding medium to high repeatabilities. The relationship between other measures of personality and acoustic signalling have been less consistent, with some studies finding a relationship between acoustic signalling and personality traits such as activity and others reporting negative or conflicting relationships (see [14] for a review). Given the ubiquitous nature of acoustic signalling in mammals, understanding the relationship between personality and vocalizations can provide valuable insights into the role of personality in social interactions.

The domestic pig (*Sus scrofa domestica*) is a highly social and vocal species which uses acoustic signals in a variety of contexts, for example in maintaining contact with other group members while foraging, in parent–offspring communication, and when they are distressed [15]. Acoustic signals of pigs tend to form a graded continuum of sound from low- to high-frequency calls of which the distinctions between call types are not clear [16]. However, pig vocalizations can be grouped into high- and low-frequency calls: squeals and screams are high-frequency calls and are produced in situations of fear and thus may function as appeasement signals or to alert conspecifics [16,17]; grunts are low-frequency calls which occur in all contexts, but are typical of foraging contexts and thus are thought to function as a contact call to indicate the location of the caller to other members of the group [15]. In domestic pigs coping styles are related to immune response [18], aggression at weaning [19] and adaptation to social isolation [20]. Furthermore, proactive pigs produce more vocalizations in response to novelty [20,21], suggesting that acoustic signalling may be repeatable; however, these studies did not investigate the repeatability of calling. Acoustic signalling in pigs has mainly been studied in relation to welfare status [22–24], and previous studies have primarily focused on vocalizations produced during short-term stressors. Yet living in a barren environment is one of the most prevalent chronic stressors in modern pig production [25] and exposure to long-term stressors may affect acoustic signalling. Environmental enrichment improves the welfare of pigs by enabling animals to perform highly motivated species-specific behaviours such as foraging [26]. Thus, we aimed to investigate the effect of environmental quality on personality and acoustic signalling in pigs by comparing individuals from different environmental treatments.

We investigated the effects of barren versus enriched housing conditions on acoustic signalling rate in individual juvenile domestic pigs, and tested for an association with personality. We assessed personality by measuring behavioural responses to two different stressful situations: a novel object test and a social isolation test. Each type of test was repeated once to allow us to assess the repeatability of behaviour within and between these contexts. We predicted that the acoustic signalling rate would be a repeatable behavioural trait and that it would be related to personality measures in pigs. Moreover, we predicted that the environmental conditions individuals are exposed to would affect vocalization rate.

## 2. Material and methods

### 2.1. Animals and housing

A total of 288 pigs over four replicates (commercial crossbreed PIC 337 (Large White × Landrace)) were weaned at four weeks of age and housed in four groups of 18, balanced for weight and sex. All pigs came from the same pre-weaning environment, a standard commercial farrowing system with the sow being confined to a farrowing crate and plastic slats as the flooring material. Two groups per replicate were housed in barren pens (l × w: 3.42 × 2.18 m) which had partially slatted concrete floors with two wooden blocks hanging from the ceiling as enrichment. Two groups per replicate were housed in enriched pens (l × w: 5.16 × 2.18 m) which had solid floors with straw bedding replenished as required and three wooden blocks hanging from the ceiling. The enriched pens had a greater space allowance per pig of 0.62 m^2^ compared with 0.41 m^2^/pig in the barren pens. The provision of the wooden blocks in both environments was necessary to adhere to the minimum enrichment provision standards for pigs in the barren environment [27]. A standard commercial diet for weaned pigs and water were available ad libitum in all pens. A lighting schedule of 12 : 12 was used with lights on at 08.00 h, and additional natural light entered the room through four windows at the front and back of the room. The ventilation and temperature were automatically controlled. As per standard husbandry practice, on entry of the pigs to the experimental room the target temperature was 28°C and this decreased by 0.5°C each day to 19°C, at which it was maintained.

A total of 72 pigs from the four replicates were tested in the current study: 24 pigs each from replicates 1 and 2, and 12 pigs each from replicates 3 and 4. On weeks 1 and 3 post-weaning, the pigs were scored based on their level of injury (to the body from aggression and tail from harmful social behaviour). To avoid bias in the two experimental groups, pigs were chosen for testing based on their total injury score from weeks 1 and 3. In each pen, the two pigs with the highest injury scores, i.e. greatest number and severity of injuries, the two pigs with the lowest injury scores, i.e. lowest number and severity of injuries, and two additional pigs chosen at random from within the pen were selected for testing (further details of injury scoring are available in the electronic supplementary material). In total, there were 34 females, 17 each from the barren and enriched environments, and 38 males, 19 each from the barren and enriched environments.

### 2.2. Personality testing

#### 2.2.1. Social isolation

A social isolation test was performed on the selected pigs, first at six weeks (test 1) and again at eight weeks of age (test 2). The test animal was removed from its home pen and walked down a short corridor to a room which contained the test pen. The test pen consisted of a concrete floor and plywood walls (l × w × h: 2.2 × 1.7 × 1.2 m) and the test individual was held here for the 3 min duration of the test. After this, the individual was removed and either taken to the novel object arena for a 5 min habituation period (after test 1) before being returned to its home pen or returned directly to its home pen (after test 2).

#### 2.2.2. Novel object test

The novel object tests were performed on the day after the social isolation tests when pigs were six weeks and eight weeks old, respectively. The order in which individuals were tested was randomized for both the novel object and social isolation tests at each age. The test pig was removed from its home pen and walked down a short corridor to the test room where it was held in the start box for 1 min. The start box (plywood walls and concrete floor: l × w × h: 1 × 1 × 1.2 m) was attached to the lower right-hand corner of the novel object arena and the pigs entered the arena from it via a sliding wooden door. The novel object arena consisted of a concrete floor and plywood walls (l × w × h: 3.6 × 2 × 1.2 m). The novel objects were a large white bucket and an orange traffic cone, both of which the pigs were unfamiliar with. Each individual received both objects and object presentation order was pseudo-randomized and counterbalanced between treatments and across tests 1 and 2, with the constraint that order was balanced across treatments. After 1 min in the start box, the sliding door was opened and the pig could enter the arena. They were allowed a maximum of 2 min to enter the arena and all animals did so within this time. Once the pig had entered the arena, the sliding door was closed behind them and the novel object was lowered into the arena from the ceiling to a distance of 10 cm from the ground, where it remained suspended by a rope for the entire test. The test began as soon as the object was in the correct position (3–5 s from the time of the sliding door being closed) and lasted a total of 5 min. When the test period was over the test pig was released from the arena and immediately returned to its home pen. The test arena was cleaned after each test, and deep-cleaned between testing of animals from different pens and at the end of each test day.

### 2.3. Vocalization recording and analysis

Vocalizations produced during all tests were recorded using a Sennheiser ME66/K6 directional microphone connected to a Marantz PMD660 solid-state audio recorder (.wav format, sample frequency: 44.1 kHz, resolution: 16 bit). To calculate acoustic signalling rates for each individual (number of vocalizations/minute), all vocalizations were visualized and counted in Avisoft SASLab Pro 5.1.21 (Avisoft Bioacoustics, Berlin, Germany). Low-frequency (less than 500 Hz) and high-frequency (greater than 500 Hz) vocalization rates were calculated separately for each individual. Owing to the fact that no high-frequency vocalizations were recorded in 60% of all tests, it was not possible to assess repeatability of high-frequency vocalization rate and so it was excluded from further analysis. Technical difficulties resulted in the loss of vocalization recordings from three novel object tests (one each for three different individuals), thus reducing our sample size to 69 when creating the mean acoustic signalling score.

### 2.4. Behavioural recording and analysis

Video recordings were made of all tests using an overhead CCTV camera in each test arena. The videos were analysed using JWatcher v. 1.0. The duration of standing, exploring the arena (sniffing, licking, biting or touching the floor/walls of the arena with nose) and the frequency of line crossing with the two front feet were recorded. To measure line crossing, each test arena was divided on screen into 9 or 12 equal sized rectangles (for social isolation and novel object arenas, respectively). These behaviours provide a measure of overall activity in the test. Additionally, for the novel object tests, latency to contact the novel object and duration of contact with the object were recorded to assess neophobia. Owing to a recording mistake, the behavioural measures for one novel object test for one individual was missing, reducing our sample size to 71 when creating the personality score.

### 2.5. Statistical analysis

Data analysis was conducted in R v. 3.0.2 (R Development Core Team 2008) and IBM SPSS v. 20 (SPSS Inc., Chicago, IL, USA). Firstly, as we did not assess personality before the pigs were placed in their respective environmental treatment groups, we investigated whether there was a difference in repeatability of behaviours between barren and enriched individuals. To do this, we ran linear models on all behaviour and vocalization variables. Each variable's value from test 1 was set as the dependent variable, with the test 2 value for that variable and environment, along with their interaction, included as fixed effects (e.g. lm(NOstand.test1 ∼ NOstand.test2 × Environment)). A non-significant interaction term would indicate that the relationship between the variable's test 1and test 2 values was not affected by environment.

We investigated individual consistency in behaviour and acoustic signalling rate from the novel object and social isolation tests using the intraclass correlation coefficient (ICC) as a measure of repeatability. Only behaviours which had significant repeatability were considered in further statistical analysis, as potential candidate measures of personality with consistency across time/contexts.

Next, we investigated if the repeatable behaviour variables recorded in both the social isolation and novel object tests were measuring the same trait. We created *z*-scores for each variable (social isolation (SI) and novel object (NO) duration standing, SI and NO duration explore, latency to approach the novel object (latency), and SI and NO low-frequency vocalization rates (hereafter ‘acoustic signalling rates’)) from the mean of tests 1 and 2 for each individual. A correlation matrix of the *z*-scores of the following variables; SI and NO standing, SI and NO exploring and latency to contact the novel object, was created to investigate the structure of the data. Chronbach's alpha tested the extent of correlation between the variables, in order to determine whether they were all measuring the same latent trait. A Cronbach's *α* ≥ 0.7 indicates that all variables are measuring the same latent trait and means that these variables can be combined to use as a potential personality measure. As items need to be directionally correlated to provide an accurate *α*-value, any positively correlated items were inverted by multiplying them by −1 before inclusion in the alpha model. Both SI exploring and NO exploring, as well as latency were positively correlated with the other variables, therefore these three variables were inverted. We termed the aggregated score produced from this analysis the ‘proactivity–reactivity (PR) index’. Chronbach's alpha also tested whether acoustic signalling rates across the novel object and social isolation tests could be combined to give an aggregate score. In a preliminary analysis, we investigated the relationship between injury score and both acoustic signalling rate and PR index score as injury score may provide some information about aggression in pigs [28]. However, no associations were found and injury score was not investigated further.

A mixed effects model was used to investigate the fixed effects of environment and sex and their interaction on PR index score, with replicate included as a random intercept to control for significant variability between replicates. PR index was squared to consider the potential for nonlinear effects, and due to problems with heteroscedasticity we used the log of PR index squared. Finally, a linear mixed effects model was used to investigate the fixed effects of personality (as measured with the PR index), sex, environment and their interactions on acoustic signalling rate, with replicate included as a random intercept to control for significant variability between replicates. Model construction began with the inclusion of all fixed effects and their interactions (PR index × sex × environment) and the random intercept, non-significant terms were then deleted using stepwise backward model selection. Model residuals were visually checked for normality to ensure they met the assumptions of the models. Significant interaction effects were further analysed using a post hoc Tukey test.

## 3. Results

In the linear models used to investigate whether there was a difference in repeatability of behaviours between barren and enriched individuals, none of the interaction terms were found to be significant (all *p* > 0.1), hence there was no evidence that repeatability was affected by environmental treatment. Acoustic signalling rates and five out of the seven behavioural variables were found to be significantly repeatable across tests at six and eight weeks of age (table 1). Of the behavioural measures, total duration of standing and total duration of exploring in both the novel object (NO) and social isolation (SI) tests, and latency to contact the NO were found to be repeatable. Chronbach's alpha for these five repeatable behavioural variables was *α* = 0.858, therefore these variables were combined by calculating the per-individual mean to create the PR index. Lower scores on this index indicate a more reactive coping style with more exploring the arena, less standing and being slower to contact the novel object. Higher scores indicate a more proactive coping style with less time spent exploring the arena, more time standing and being quicker to contact the novel object. Acoustic signalling rate was also found to be highly repeatable within both the SI tests and the NO tests. The alpha for the acoustic signalling rates from the SI and NO tests was *α* = 0.843, thus these variables were combined to create a mean acoustic signalling rate from across tests.

**Table 1.** Repeatability estimates from intraclass correlation coefficient (ICC) for acoustic signalling and behavioural variables from the novel object (NO) and social isolation (SI) tests.

vocalization/behaviour	ICC (1)	lower CI	upper CI	*p*-value
SI acoustic signalling rate	0.579	0.403	0.714	<0.001
NO acoustic signalling rate	0.484	0.281	0.645	<0.001
duration stand in NO	0.359	0.142	0.544	0.001
duration stand in SI	0.286	0.059	0.485	0.007
duration explore in SI	0.482	0.283	0.642	<0.001
duration explore in NO	0.461	0.259	0.625	<0.001
latency to contact NO	0.292	0.067	0.488	0.006
duration investigating NO	0.020	−0.210	0.249	0.432
line cross frequency NO	−0.108	−0.349	0.147	0.797

**Figure 1.**
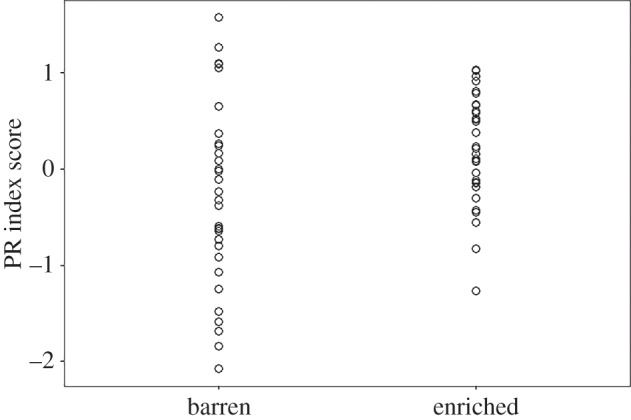
Distribution of raw personality (PR index) scores for pigs in barren and enriched environments.

The model using log PR index squared revealed that PR index score was affected by environment with individuals from the enriched environment (raw mean ± s.e.m.: 0.215 ± 0.096) scoring higher on the index than pigs from the barren environment (raw mean ± s.e.m.: −0.232 ± 0.166), suggesting that the pigs from the enriched environment are more proactive (*β* ± s.e. = −0.26 ± 0.09, *t*_63_ = −2.74, *p* = 0.008; figure 1). There was no significant effect of sex on PR index score (*β* ± s.e. = 0.002 ± 0.1, *t*_62_ = 0.02, *p* = 0.98), nor was there a significant effect of the interaction between sex and environment in PR index score (*β* ± s.e. = −0.11 ± 0.2, *t*_62_ = −1.45, *p* = 0.59).

**Figure 2.**
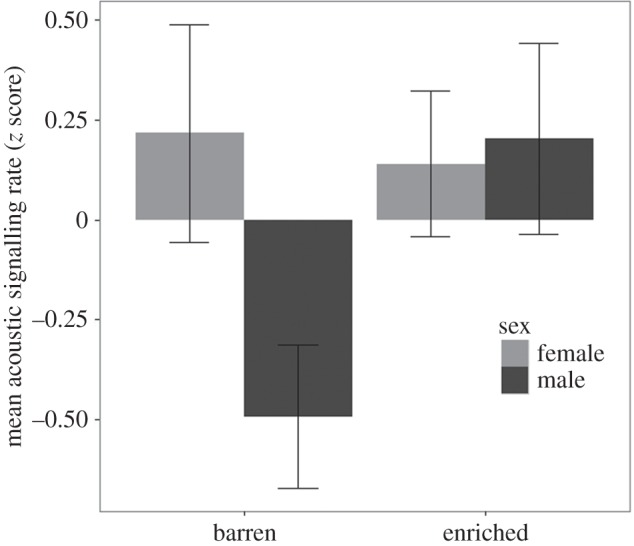
Mean ± s.e. acoustic signalling rate in males and females from the barren and enriched environments.

The final model for acoustic signalling rate included the fixed effect PR index score, the interaction term sex × environment and replicate as a random intercept. Acoustic signalling rate was predicted by PR index, with more proactive individuals signalling at a higher rate than reactive individuals (table 2). There was also an interaction effect of environment and sex on acoustic signalling rate (table 2 and figure 2). Males from barren environments had a significantly lower signalling rate than males from enriched environments, and significantly lower than the signalling rates of females from barren environments (all post hoc Tukey tests, *p* < 0.05). Females from enriched environments showed a non-significant tendency for higher signalling rates than males from barren environments (post hoc Tukey test, *p* = 0.058). There was no significant difference in signalling rates between females from the two different environments (post hoc Tukey test, *p* > 0.05).

**Table 2.** Effects of personality, environment and sex on acoustic signalling in pigs, *Sus scrofa domestica.* Linear mixed effects model for acoustic signalling rate (*n* *=* 68).

variable	estimate	s.e.	*t*-value	*p*-value
intercept	0.418	0.260	1.607	0.113
PR index (personality)	0.531	0.110	4.822	<0.001
environment	−0.277	0.244	−1.130	0.263
sex	−0.732	0.243	−3.017	0.037
environment : sex	0.708	0.330	2.147	0.036

## 4. Discussion

Acoustic signals play a central role in interactions among individuals and the present results demonstrate consistent individual differences in signalling behaviour in a highly social mammal. Here we show that acoustic signalling rate (hereafter acoustic signalling) covaries with other behavioural measures of personality in juvenile domestic pigs. Acoustic signalling was the most highly repeatable behaviour of the behaviours measured and acoustic signalling in the social isolation context was highly correlated with acoustic signalling in the novel object context. Acoustic signalling correlated with the multi-behaviour personality index for PR. Thus, acoustic signalling may provide a useful and easily measured indicator of personality in juvenile pigs. This result is in broad agreement with studies in several bird species, where it has been shown that there is a positive relationship between exploration score and signalling rate [29–31]. As acoustic signalling rate varied between males in different environments, but this effect was not found on personality, caution should be taken if using acoustic signalling as a sole measure of personality in juvenile pigs.

As acoustic signals contain information about the signaller, these results imply that personality may impact on the nature of interactions within social groups. From our results, acoustic signalling is strongly linked to personality, thus it may be possible that receiving individuals could detect information about the signaller's personality through its vocalizations. In humans, vocalization and personality characteristics are linked, and vocal cues are used to assess the personality of conspecifics [32–34]. Detecting a conspecific's personality type from information encoded in the variation of acoustic signalling rate may be advantageous in a variety of contexts. For example, proactivity is related to aggression, with more proactive individuals displaying higher levels of aggression [35,36]. The higher rate of acoustic signalling in these animals may aid opponent assessment during agonistic interactions [37]. We found no association with injury score and either PR index score or acoustic signalling rate in our preliminary analysis, and thus did not include injury scores in further analysis. However, injury score is not a direct measure of aggression in pigs, therefore further research is necessary to investigate the relationship between aggression, personality and acoustic signalling in this species. Recently, it has been suggested that sexual selection may play a role in maintaining personality differences between individuals [38]. There is some evidence that females may choose a partner based on personality [39] and that there are potential fitness benefits related to assortative mating [40,41]. It is unclear how females may assess personality of a potential mate but the present findings raise the possibility that acoustic signalling could play a role. One possibility to explain the underlying relationship between acoustic signalling and personality is that vocalizations reflect the emotional state of the caller [2,42], and they have been correlated with neuroendocrine responses to stress [43]. Evidence from coping styles research [44] links neuroendocrine response to consistent individual behavioural responses, thus the relationship between acoustic signalling and personality may be mediated by neuroendocrine factors.

In this study, several behaviours across all tests were repeatable and highly correlated, which is consistent with the proactive and reactive behavioural types found in coping styles. Animals that were quicker to contact the novel object spent less time exploring the test arena and spent more time standing, which is consistent with a more proactive coping style [36]. Previous research into pig personalities has produced conflicting results (e.g. [18,45–47]). These conflicts may result from the use of different methods of categorizing personality types, with some using a binary approach. Individuals may be more likely to fall somewhere along a continuum, with few individuals behaving purely reactively or proactively [48]. Whether the correlations in behaviour found in this study alter with age and whether they are associated with other variables, such as aggression and behavioural flexibility, as has previously been reported [7,49], remains to be shown. Although we aimed to measure the repeatability of behavioural responses to two different stressors, i.e. a novel object and social isolation, both test types were conducted in isolation and, therefore, both may be a reaction to social isolation. Alternatively, as both tests involved some aspect of novelty (novel object and novel environment in the social isolation test), it is possible that the response to novelty was the main driver behind the behaviours we recorded. In either case, it would be useful in the future to measure these behaviours and the relationships between them in more varied situations to investigate if the trait found here is robust.

Environmental quality influenced personality score, with individuals from the enriched environment being more proactive than individuals from the barren environment. This result is in line with the findings on spiders [50], in which spiders that were kept in an enriched environment displayed behavioural traits which were typical of a more proactive coping style than spiders that were kept in a barren environment. However, we also found that pigs from barren environments were more likely to show extremes in personality scores compared with pigs in the enriched environment. Early life experiences affect personality later in life, for example lambs reared in isolation were more ‘withdrawn’ in behavioural tests than lambs reared with their peers or lambs reared with their mothers [51]. The findings of the present study are in keeping with these results; however, few studies have investigated the effects of chronic environmental stressors on personality score, therefore this topic requires further research to understand the mechanisms behind our results. The design of this study aimed to maximize the difference between the environments while keeping the differences relevant to standard farming practices, thus we combined extra space allowance and straw provision to create the enriched environment. Thus, we are unable to specify which factor may have contributed most to the difference between environments.

Signalling rate in males, but not females, was influenced by the environmental treatment, suggesting that males are affected by a chronic stressor differently from females. The lack of a difference in personality score between the sexes rules out the possibility that sex differences in personality could explain this result. The low vocalization rate in barren male pigs may be indicative of a reduced welfare status induced by living in a barren environment. In general, increased vocalization rates are associated with reduced welfare status, especially during acute stressors [16,52]. Nonetheless, chronic stressors may give rise to different behavioural responses [53], and little is known about the impacts of chronic stress on vocalization in mammals, or indeed whether there are sex specific differences in these responses. In long-term isolated chicks, a reduced vocalization rate was associated with a depressive-like state of ‘learned helplessness’ [53]. Furthermore, sex differences in stress response have been reported in rats, where males show more anxiety behaviour than females in an elevated plus maze test following a 7-day restraint stressor [54]. However, we cannot rule out the possibility that vocalization rate within the social isolation and novel object tests could be reflective only of the pigs' states while in those tests, and not of their state outside those conditions. Research incorporating more measures of anxiety and/or depression and also including physiological measurements will be necessary to elucidate the interpretation of reduced vocalization rates in relation to welfare status.

In conclusion, we found that acoustic signalling rate is repeatable and related to personality in a highly social mammal. Additionally, we found that the acoustic signalling rate among males varied significantly between barren and enriched environments. Understanding how acoustic signalling is related to personality will help elucidate how personality affects communication and thus its role in the evolution of social behaviour and group dynamics in gregarious species.

## Supplementary Material

ESM1: Injury scoring scales.docx

## Supplementary Material

ESM2: Mixed models R code.docx

## Supplementary Material

ESM3: Data.csv

## Supplementary Material

EMS4: Correlation matrix of behavioural variable z-scores.docx

## Data accessibility

All data supporting this study have been uploaded as part of the electronic supplementary material.
