# Supplementary material for: Acoustic signalling reflects personality in a social mammal
Source: R Soc Open Sci. 2016 Jun 29;3(6):160178. doi: 10.1098/rsos.160178 (PMC4929910; doi:10.1098/rsos.160178)
Supplement: ESM1: Injury scoring scales.docx [file rsos160178supp1.docx]

Supplement 1: Injury scoring methods and scales

On weeks 1 and 3 of each replicate, physical indicators of health were recorded for each individual pig. On each scoring day, all pens of pigs were observed and every pig within each pen was observed sequentially by one experimenter (KG). To score the pigs, KG entered the pen to observe injury levels, which involved slowly walking around the pigs and stating the injury scores to the second experimenter (MF) who remained outside the pen recording the scores. Each body part of the pig was assessed for injuries separately. Areas assessed were: left ear, right ear, snout, left shoulder, right shoulder, front legs, back legs, left flank, right flank, left hindquarter, right hindquarter and back. Level of injury was scored on a 6-point scoring system modified from Conte et al. (2012):

0 - No injuries

1 - One small superficial lesion

2 - More than one small, superficial lesion; or just one red (deeper than score 1) but still superficial lesion

3 - One or several big and deep lesions

4 - One very big, deep and red lesion or many deep, red lesions

5 - Many very big, deep and red lesions covering the skin area

Injuries to the tail that were caused by tail biting were also assessed using a 6-point scoring system adapted from Kritas & Morrison (2007):

0 - No evidence of tail-biting

1 - Healed or mild lesions

2 - Evidence of chewing or puncture wounds, but no evidence of swelling

3 - Evidence of chewing or puncture wounds with swelling and signs of possible infection

4 - Partial loss of the tail

5 - Total loss of tail up to the hindquarters

Each individual’s injury score comprised the sum of scores from the entire body and individual score from weeks 1 and 3 were summed per individual to give the final injury score on which individuals were selected for testing.

References:

Conte, S., Lawlor, P. G., O'Connell, N. & Boyle, L. A. 2012 Effect of split marketing on the welfare, performance, and carcass traits of finishing pigs. *J. Anim. Sci*. **90**, 373-380.

Kritas, S. K. & Morrison, R. B. 2007. Relationships between tail biting in pigs and disease, lesions and condemnations at slaughter. *Vet. Rec*. **160**, 149-152.
