# Supplementary material for: Acoustic signalling reflects personality in a social mammal
Source: R Soc Open Sci. 2016 Jun 29;3(6):160178. doi: 10.1098/rsos.160178 (PMC4929910; doi:10.1098/rsos.160178)
Supplement: ESM2: Mixed models R code.docx [file rsos160178supp2.docx]

Supplement 2: R code for mixed models

mod1 <- lme(meanAcousticSig ~ PRindex*sex*environ, data=st1, random= ~1|rep, method= "ML")

summary(mod1)

mod2 <- lme(meanAcousticSig ~ (PRindex+sex+environ)^2, data=st1, random= ~1|rep, method= "ML")

summary(mod2)

mod3 <- lme(meanAcousticSig ~ PRindex+sex+environ + environ:sex + PRindex:environ, data=st1, random= ~1|rep, method= "ML")

summary(mod3)

mod4 <- lme(meanAcousticSig ~ PRindex+sex+environ + environ:sex, data=st1, random= ~1|rep, method= "ML")

summary(mod4)

## Post-hoc on the interaction term

st1$SHD<-interaction(st1$environ,st1$sex)

mod4<-lme(meanAcousticSig~-1+SHD, data=st1, random=~1|rep)

library(multcomp)

summary(glht(mod4,linfct=mcp(SHD="Tukey")))

## Model with log PR index squared to control for non-linear effects

prsq1 <- lme(log(PRsq+1) ~ sex * environ, data=st1, random= ~1|rep, method= "ML")

prsq2 <- lme(log(PRsq+1) ~ sex + environ, data=st1, random= ~1|rep, method= "ML")

prsq3 <- lme(log(PRsq+1) ~ environ, data=st1, random= ~1|rep, method= "ML")
