# Supplementary material for: Acoustic signalling reflects personality in a social mammal
Source: R Soc Open Sci. 2016 Jun 29;3(6):160178. doi: 10.1098/rsos.160178 (PMC4929910; doi:10.1098/rsos.160178)
Supplement: EMS4: Correlation matrix of behavioural variable z-scores.docx [file rsos160178supp4.docx]

Supplement 4: Pearson correlation matrix of the z-scores for the behavioural variables recorded in the Social isolation (SI) and Novel object (NO) tests. Note: z-scores for each variable were created from the mean of tests 1 and 2 for each individual.

|  | | | | | | | | |
| --- | --- | --- | --- | --- | --- | --- | --- | --- |
|  | | Latency | NO explore | NO stand | NO acoustic signalling | SI explore | SI stand | SI acoustic signalling |
| Latency | Pearson Correlation | 1 | .408^**^ | -.162 | -.419^**^ | .447^**^ | -.312^**^ | -.412^**^ |
|  | Sig. (2-tailed) |  | .000 | .175 | .000 | .000 | .008 | .000 |
|  | N | 72 | 72 | 72 | 69 | 71 | 71 | 72 |
| NO explore | Pearson Correlation | .408^**^ | 1 | -.860^**^ | -.531^**^ | .636^**^ | -.622^**^ | -.398^**^ |
|  | Sig. (2-tailed) | .000 |  | .000 | .000 | .000 | .000 | .001 |
|  | N | 72 | 72 | 72 | 69 | 71 | 71 | 72 |
| NO stand | Pearson Correlation | -.162 | -.860^**^ | 1 | .340^**^ | -.532^**^ | .574^**^ | .295^*^ |
|  | Sig. (2-tailed) | .175 | .000 |  | .004 | .000 | .000 | .012 |
|  | N | 72 | 72 | 72 | 69 | 71 | 71 | 72 |
| NO acoustic signalling | Pearson Correlation | -.419^**^ | -.531^**^ | .340^**^ | 1 | -.431^**^ | .330^**^ | .728^**^ |
|  | Sig. (2-tailed) | .000 | .000 | .004 |  | .000 | .006 | .000 |
|  | N | 69 | 69 | 69 | 69 | 68 | 68 | 69 |
| SI explore | Pearson Correlation | .447^**^ | .636^**^ | -.532^**^ | -.431^**^ | 1 | -.927^**^ | -.589^**^ |
|  | Sig. (2-tailed) | .000 | .000 | .000 | .000 |  | .000 | .000 |
|  | N | 71 | 71 | 71 | 68 | 71 | 71 | 71 |
| SI stand | Pearson Correlation | -.312^**^ | -.622^**^ | .574^**^ | .330^**^ | -.927^**^ | 1 | .401^**^ |
|  | Sig. (2-tailed) | .008 | .000 | .000 | .006 | .000 |  | .001 |
|  | N | 71 | 71 | 71 | 68 | 71 | 71 | 71 |
| SI acoustic signalling | Pearson Correlation | -.412^**^ | -.398^**^ | .295^*^ | .728^**^ | -.589^**^ | .401^**^ | 1 |
|  | Sig. (2-tailed) | .000 | .001 | .012 | .000 | .000 | .001 |  |
|  | N | 72 | 72 | 72 | 69 | 71 | 71 | 72 |
| **. Correlation is significant at the 0.01 level (2-tailed). *. Correlation is significant at the 0.05 level (2-tailed). | | | | | | | | |
